# Atrial fibrillation burden and clinical outcomes following BTK inhibitor initiation

**DOI:** 10.1038/s41375-024-02334-3

**Published:** 2024-08-17

**Authors:** John Alan Gambril, Sanam M. Ghazi, Stephen Sansoterra, Mussammat Ferdousi, Onaopepo Kola-Kehinde, Patrick Ruz, Adam S. Kittai, Kerry Rogers, Michael Grever, Seema Bhat, Tracy Wiczer, John C. Byrd, Jennifer Woyach, Daniel Addison

**Affiliations:** 1 Department of Internal Medicine, Ohio State University Medical Center, Columbus, OH USA; 2 Cardio-Oncology Program, Division of Cardiology, The Ohio State University Medical Center, Columbus, OH USA; 3 Division of Hematology, The Ohio State University, Columbus, OH USA; 4 Department of Pharmacy, James Cancer Hospital and Solove Research Institute, Columbus, OH USA; 5 Department of Medicine, University of Cincinnati, Cincinnati, OH USA; 6 Division of Cancer Prevention and Control, Department of Internal Medicine, College of Medicine, The Ohio State University, Columbus, OH USA

**Keywords:** Chronic lymphocytic leukaemia, Adverse effects, Molecularly targeted therapy

## Abstract

Bruton’s tyrosine kinase inhibitors (BTKi) have dramatic efficacy against B-cell malignancies, but link with cardiotoxicity, including atrial fibrillation (AF). Burden, severity, and implications of BTKi-related AF are unknown. Leveraging a large-cohort of consecutive B-cell malignancy patients initiated on BTKi from 2009–2020, we identified patients with extended ambulatory rhythm monitoring. The primary outcome was AF burden after BTKi-initiation. Secondary outcomes included ventricular arrhythmia burden and other arrhythmias. Observed incident-AF rates and burden with next-generation BTKi’s were compared to ibrutinib. Multivariable regression defined association between rhythm measures and major adverse cardiac events (MACE), and mortality. There were 98 BTKi-treated patients [38.8% next-generation BTKi’s, 14.3% prior-AF], with 28,224 h of monitoring. Median duration BTKi-use was 34 months. Over mean duration 12 days monitoring, 72.4% developed arrhythmias (16.3% incident-AF, 31.6% other SVTs, 14.3% ventricular tachycardia). 14.3% had high AF-burden. AF-burden was similar between ibrutinib and next-generation BTKi’s. No single antiarrhythmic-therapy prevented BTKi-related AF. However, antiarrhythmic initiation associated with reduction in arrhythmic burden (*P* = 0.009). In a multivariable model accounting for traditional cardiovascular risk factors, prior-AF associated with increased post-BTKi AF-burden. In follow-up, high AF burden associated with MACE (HR 3.12, *P* = 0.005) and mortality (HR 2.97, *P* = 0.007). Among BTKi-treated patients, high AF burden prognosticates future MACE and mortality risk.

## Introduction

Bruton’s tyrosine kinase inhibitors (BTKi’s) have dramatic efficacy against B-cell malignancies and are now approved as indefinite therapy in those without disease progression or intolerance [1–3]. Next-generation BTKi’s (e.g., acalabrutinib, pirtobrutinib) show less cardiac activity than the first-generation BTKi ibrutinib [2–5]. However, recent unexplained cases of serious arrhythmias, including atrial fibrillation (AF) and ventricular arrhythmias have been reported in next-generation BTKi’s [6, 7]. With ibrutinib, similar insidious arrhythmic events were not appreciated until well after initial clinical availability [6–12].

To date, the assessment and understanding of cardiotoxicity with BTKi’s has primarily focused on evaluation in a binary fashion (present vs. absent) [13]. However, growing evidence suggests the clinical effects of cardiotoxic AF and other arrhythmic events may not be well captured by the presence or absence of these cardiotoxic events alone [14, 15]. In available longitudinal non-cancer cohorts, elevated burdens or patterns of AF as measured by continuous, rather than 10 s, electrocardiographic (ECG) monitors strongly correlate to long-term consequences of incident AF. In cancer patients, anecdotal reports suggest potentially substantial increase in AF burden and duration with ibrutinib [15]. Yet, whether this pattern holds with other BTKi’s and the degree to which high BTKi-related AF burden has ramifications on the development of other serious events remains unknown.

## Methods

### Study population

Leveraging a large and contemporary US-based Comprehensive Cancer Center cohort of consecutive patients treated with ibrutinib, acalabrutinib, and other next-generation BTKi’s (e.g., pirtobrutinib, nemtabrutinib) from 2009 to 2020 following Institutional Review Board approval, we explored the burden of AF. Study patients included adults ≥18 years old treated with BTKi for a hematologic malignancy who had ambulatory cardiac rhythm monitoring performed for suspected cardiotoxicity. Patients with incomplete medical records for the variables of interest or fewer than 7 days of BTKi use were excluded. We manually searched all subject charts for all ambulatory telemetry monitors inclusive of the reporting of potential AF events and worn within the time of BTKi initiation to 6 months after cessation. Ambulatory cardiac rhythm monitors included traditional Holter monitors, event monitors, mobile cardiac telemetry, and implantable loop recorders [16, 17]. Cardiovascular rhythm monitor analyses were performed blinded to clinical information.

AF burden was defined as the proportion of time a patient was observed to be in AF during a monitoring period, expressed as the percentage (%) of time in AF over a 24 h period for consistency, in line with established cardiac and rhythm society consensus definitions [18]. We also assessed the burden of ventricular arrhythmia events, including premature ventricular contractions, non-sustained ventricular tachycardia (NSVT), sustained ventricular tachycardia (VT), and ventricular fibrillation, as well as high-degree atrioventricular blocks. Arrhythmia episodes were graded using the Common Terminology Criteria for Adverse Events v5.0, followed by adjudication by independent cardiologists [19]. Arrhythmia-associated symptoms included chest pain, palpitations, dizziness, syncope, heart failure symptoms, and sudden cardiac death. Moreover, we manually searched all patient charts for incident major adverse cardiovascular events (MACE), defined as stroke, congestive heart failure (CHF), myocardial infarction (MI), symptomatic AF- or VT-related hospital admission, and cardiovascular or sudden cardiac death.

### Outcomes

The primary outcome was the presence of high (≥10%) AF burden after BTKi initiation. The secondary outcome was the presence of increased (≥5%) burden of any arrhythmia, including supraventricular and ventricular arrhythmias, high-degree atrioventricular block, or need for new pacemaker placement after BTKi initiation. We also explored the relation between AF burden and the occurrence of MACE and mortality after BTKi initiation. Follow-up began from the time of BTKi initiation. Naranjo Probability Scores were estimated for AF, VAs, and MACE, to determine the likelihood of BTKi association, with a score of ≥6 suggestive of at least a probable association. Furthermore, we explored the effects of baseline antiarrhythmic use on the avoidance and control of high AF burden.

### Statistical analysis

Descriptive statistics were used to summarize patient characteristics, using mean +/−, standard deviation (SD), or median (interquartile range, IQR) for continuous variables as appropriate, and frequency counts with percentages for categorical variables. Data for continuous variables were then compared utilizing unpaired Student’s t-test or Wilcoxon rank-sum test as appropriate for each variable. Categorical data were compared utilizing chi-squared test. To further understand the relationship of AF burden to the occurrence of MACE, we constructed cumulative incidence curves, stratified according to degree of AF burden (<5, 5–9.9, and ≥10%) after BTKi initiation.

For the assessment of AF burden, univariate models were created using potential risk factors followed by multivariate models. Univariate and multivariate modeling was used to determine the association between baseline covariates, AF burden, MACE, and all-cause mortality, respectively. Receiver operator curve (ROC) curves were also generated and integrated to determine an appropriate cutoff for high AF burden relating to subsequent MACE risk [20]. Time-to-event analysis methods were used to evaluate differences in probability of all-cause mortality and MACE between high and low AF burden groups utilizing Kaplan-Meier survival analysis and the log-rank test. Univariable and multivariable Fine and Gray regression analysis accounting for competing risks of BTKi discontinuation or death were also performed to determine the association between baseline covariates and outcomes. Cumulative incidence estimates for outcomes were estimated, and incidence curves were subsequently generated. Multivariate Cox proportional hazards regression was then used to assess the association of higher AF burden and patient-specific covariates with MACE and all-cause mortality following BTKi therapy initiation. To avoid overfitting the Cox model, we chose to include one covariate for every 10 outcome events that occurred. All variables with *P* < 0.10 in univariate modeling were initially included in the multivariate models, and backward selection was used to sequentially (stepwise) remove variables with *P* > 0.05 from the final model. A similar modeling approach was applied for MACE, with the primary comparison of patients who developed high AF burden with patients who had no or lower AF. Low AF burden was defined as <5% of all heartbeats being AF across a 24 h period, in line with standard definitions [18]. These variables were included in the multivariable model for MACE and all-cause mortality. Landmark analysis, excluding AF events within 30 days of monitor initiation, was also performed to further delineate the effect of index BTKi-arrhythmia burden on long-term MACE risk.

To better understand comparative effects between first (ibrutinib) and next-generation BTKi’s, observed rates of AF burden were compared with available next-generation rates (e.g., ibrutinib vs. acalabrutinib; ibrutinib vs. pirtobrutinib). Similarly, comparisons were made for PVC and other arrhythmia burdens. The statistical significance of these models was then assessed via two-tailed t-test with *P* < 0.05. In addition, subgroup analysis among patients without prior AF or VT was performed. Observed AF burden rates were compared to ibrutinib-associated rates via contemporaneous data, using calculated relative risks (RRs). Comparative rates with other emerging BTKi’s (e.g., pirtobrutinib, nemtabrutinib) were also explored [21] Data was analyzed using STATA version 17 (StataCorp, College Station, TX).

## Results

Overall, 98 patients with ambulatory rhythm monitoring during treatment were identified (Supplemental Fig. 1), with 28,224 h of ambulatory monitoring available. The mean age was 68.5 ± 8.9 years (range 37–94 years), and 27.6% of patients were female. Most (97.4%) had chronic lymphocytic leukemia (CLL), and all had Eastern Cooperative Oncology Group (ECOG) performance status of 0–2. Thirty-eight (38.8%) patients were treated with next-generation BTKi therapies (e.g., acalabrutinib, pirtobrutinib); 14 (14.3%) had baseline AF at the time of rhythm monitor initiation, including five with known BTKi-associated arrhythmias. Among those on next-generation BTKi’s, 36.8% were previously treated with ibrutinib, and 10.5% received non-covalent (reversible) BTKi’s. In those transitioned between drugs, the mean time from ibrutinib cessation to initiation of next-generation BTKi therapy was 12.7 months (median 4.1 months). Median duration of BTKi use at the time of rhythm monitor was 42.0 months. The mean duration of monitoring was 12.0 days. Additional baseline characteristics are as described in Table 1, and Supplemental Tables 1–2. The most common indications for monitoring were palpitations, suspected AF, abnormal office ECG, and syncope (Supplemental Table 3).

**Table 1:** Baseline characteristics.

Characteristic	Total (*n* = 98)	Next-generation BTKi (*n* = 38)	Ibrutinib (*n* = 60)	*P*-value
BTK Inhibitor, *n* (%)				
Acalabrutinib	34 (34.7)	34 (89.5)	–	–
Ibrutinib	60 (61.2)	–	60 (100)	
Nemtabrutinib (ARQ-531)	3 (3.1)	3 (7.9)	–	
Pirtobrutinib (LOXO 305)	1 (1.0)	1 (2.6)	–	
Age (years)	68.5 (8.9)	67.7 (8.3)	69.0 (9.2)	0.49
Sex, *n* (%)				
Male	71 (72.4)	29 (76.3)	42 (70.0)	0.50
Female	27 (27.6)	9 (23.7)	18 (30.0)	
BMI (kg/ m^2^), mean (SD)	28.5 (6.3)	28.5 (5.8)	28.56 (6.59)	0.94
Other baseline traditional AF risk factors, *n* (%)				
CHF	3 (3.1)	1 (2.6)	2 (3.3)	0.84
Valvular disease	4 (4.1)	1 (2.6)	3 (5.0)	0.56
Hypertension	52 (53.1)	24 (63.2)	28 (46.7)	0.11
Hyperlipidemia	34 (34.7)	15 (39.5)	19 (31.7)	0.43
DM	18 (18.4)	5 (13.2)	13 (21.7)	0.29
MI	7 (7.1)	2 (5.3)	5 (8.3)	0.57
OSA	17 (17.3)	7 (18.4)	10 (16.7)	0.82
CKD	18 (18.4)	3 (7.9)	15 (25.0)	0.030
Any prior arrhythmia	27 (27.6)	13 (34.2)	14 (23.3)	0.24
Prior AF	14 (14.3)	9 (23.7)	5 (8.3)	0.034
Prior SVT	8 (8.2)	2 (5.3)	6 (10.0)	0.40
Prior VA	4 (4.1)	2 (5.3)	2 (3.3)	0.64
Prior BTKi-associated arrhythmia	5 (5.1)	5 (13.2)	0 (0.0)	0.004
Smoking status, *n* (%)				
Never	56 (57.1)	20 (52.6)	36 (60.0)	0.47
Former (or current)	42 (42.9)	18 (47.4)	24 (40.0)	–
Primary malignancy, *n* (%)				
CLL	86 (87.8)	37 (97.4)	49 (81.7)	0.021
MCL	6 (6.1)	0 (0.0)	6 (10.0)	0.044
Other^a^	6 (6.1)	1 (2.6)	5 (8.3)	–
Rai stage, mean (SD)	2.7 (1.4) (*n* = 79)	2.2 (1.4) (*n* = 35)	3.02 (1.32) (*n* = 44)	0.0102
Baseline ECOG performance status, mean (SD)	0.5 (0.6)	0.6 (0.5)	0.47 (0.59)	0.28
Number of prior anticancer therapies, median (IQR)	2 (1–3)	2 (0–3)	2 (1–3)	0.11
Concomitant chemotherapy, *n* (%)	30 (30.7)	12 (31.6)	18 (30.0)	0.87
Prior chemotherapy, *n* (%)	59 (60.2)	17 (44.7)	42 (70.0)	0.013
Prior monoclonal antibody, *n* (%)	70 (71.4)	20 (52.7)	50 (83.3)	0.001
Prior BTKi therapy, *n* (%)	16 (16.3)	14 (36.7)	2 (3.3)	<0.001
Prior immunomodulatory therapy, *n* (%)	14 (14.3)	5 (13.2)	9 (15.0)	0.80
No prior anticancer therapies, *n* (%)	4 (4.1)	0 (0.0)	4 (6.7)	0.10
Total duration of (any) BTKi therapy at time of rhythm monitor, months, mean (SD)	42.0 (28.7)	50.4 (28.9)	36.8 (27.4)	0.022
Duration of “Current” BTKi therapy at time of rhythm monitor, months, mean (SD)	37.0 (27.2)	38.1 (26.7)	36.3 (27.5)	0.75
LA volume index ml/m^2^, mean (SD)	19.4 (12.5) (*n* = 14)	22.7 (12.6) (*n* = 8)	15.0 (10.7) (*n* = 6)	0.29
LVEF (%)	60.6 (5.6) (*n* = 49)	62.0 (4.0) (*n* = 25)	59.2 (6.7) (*n* = 24)	0.09
Baseline cardiac medications				
Beta-blocker	20 (20.4)	9 (23.7)	11 (18.3)	0.55
Calcium channel blocker	13 (13.3)	7 (18.4)	6 (10.0)	0.23
Amiodarone	1 (1.0)	0 (0.0)	1 (1.7)	0.42
Other anti-arrhythmic	1 (1.0)	0 (0.0)	1 (1.7)	0.42
Prior AF ablation	5 (5.1)	3 (7.9)	2 (3.3)	0.32
Care team				
Cardio-oncologist	20 (20.4)	10 (26.3)	10 (16.7)	0.25
Electrophysiologist	28 (28.6)	9 (23.7)	19 (31.7)	0.39
Any cardiologist	89 (90.1)	31 (81.6)	58 (96.7)	0.012

*AF* atrial fibrillation, *BMI* body mass index, *BTKi* Brutons tyrosine kinase inhibitor, *CLL* chronic lymphocytic leukemia, *CHF* congestive heart failure, *CKD* chronic kidney disease, *DM* diabetes mellitus, *ECOG* eastern cooperative group, *IQR* interquartile range, *LA* left atrial, *LVEF* left ventricular ejection fraction, *MCL* mantle cell lymphoma, *MI* myocardial infarction, *OSA* obstructive sleep apnea, *SD* standard deviation, *SVT* supraventricular tachycardia, *VA* ventricular arrhythmia.

^a^Diffuse large B-cell lymphoma, follicular lymphoma, hairy cell leukemia, and marginal zone lymphoma.

### AF burden and other rhythms

At a median BTKi treatment duration of 42.0 months (IQR 21.0–63.5 months; range 1.1–115.4 months), 14.3% of patients developed high AF burden, in those with available rhythm monitoring. The overall median AF burden was 8.6% among those patients with paroxysmal disease, with 6.2% seeing continuous AF. A ≥ 50% AF burden was observed in 8.2% of patients. Among those without baseline AF, 19.0% developed incident (new) AF, with a median AF burden of 10.0%. Within 1 year of BTKi-initiation, the median AF burden was 10.0% in those incident AF events. In those with baseline AF, the median AF burden was 10.0%. Fourteen (14.3%) patients had high AF burden (≥10%), and two (2.0%) had a moderate (5.0–9.9%) AF burden. Of the patients with significant (mainly symptomatic) PVCs, the median PVC burden was 6.0% (with a mean of 8.1%). Elevated VA burden was observed in 14 (14.3%) patients. Arrhythmias of any kind were detected in 71 (72.4%) patients; with most of probable or definite BTKi-association. Twenty-three (23.5%) patients required drug switch or treatment cessation due to arrhythmic (or symptom) burden.

In univariate analysis, only prior AF trended towards association with high AF burden, with an odds ratio of 3.8 (*P* = 0.056; Supplemental Table 4). There was no relationship between BTKi treatment duration and AF burden. However, in multivariable modeling, prior AF [hazard ratio (HR) 4.47, *P* = 0.04] remained associated with high AF burden (Supplemental Table 5). These effects remained consistent even after adjusting for primary malignancy. Similar findings were noted using a cutoff of 5% for AF burden. There was no difference in the presence of incident AF, high AF, or PVC burden, by preceding use of standard anti-arrhythmic (Supplemental Table 6).

### First versus next-generation BTK inhibitors

**Fig. 1:**
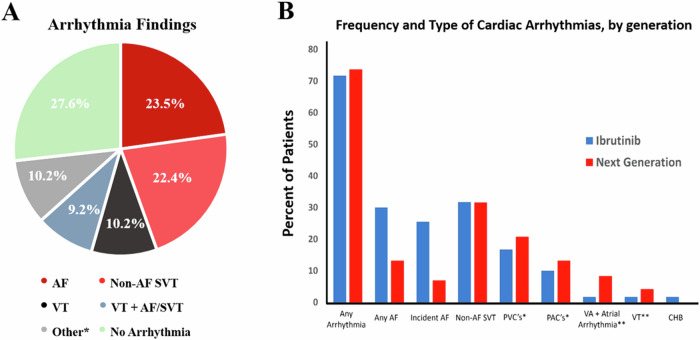
Distribution of arrhythmias detected by extended ambulatory rhythm monitors. **A** Proportions of arrhythmia findings on ambulatory rhythm monitoring in 98 patients with suspected BTK inhibitor cardiotoxicity. *Includes ≥1% premature atrial contractions, ≥1% premature ventricular contractions, and/or complete heart block. In cases of overlap of arrhythmias, AF is preferentially shown here. **B** Frequency and type of cardiac arrhythmias observed upon ambulatory (≥24 h) rhythm monitoring in patients with probable or definite BTKi-cardiotoxicity. *Includes those with symptomatic or ≥1% burden premature ventricular or atrial contractions; excludes those with asymptomatic rare premature atrial contractions, premature ventricular contractions, and next-generation patients previously exposed to ibrutinib. **Includes those with ≥10 beats of ventricular tachycardia (VT). AF atrial fibrillation; CHB complete heart block; PACs premature atrial contractions; PVCs premature ventricular contractions; SVT supraventricular tachycardia; VA ventricular arrhythmia; VT ventricular tachycardia.

**Fig. 2:**
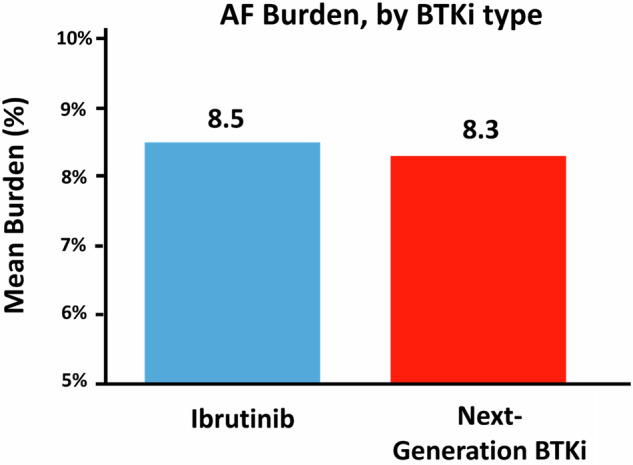
Comparative burden of AF between first (ibrutinib) and next-generation BTKIs among patients with suspected cardiotoxicity. AF atrial fibrillation, BTKi Bruton’s tyrosine kinase inhibitor.

There was no difference in the indications for or duration of rhythm monitoring by generation of BTKi therapy (10.4 days for next-generation vs.13.1 days for ibrutinib; Supplemental Tables 3 and 7A). Arrhythmia of any kind was seen in 73.7% of next-generation users and 71.7% of ibrutinib users (Fig. 1). AF was seen in 13.2% of next-generation users and 30.0% of ibrutinib users. Of those without history of AF, incident AF was less common in next-generation users than ibrutinib users (6.9% vs 25.5%, *P* = 0.039). There was no difference in the mean burden of AF (8.5% ibrutinib vs. 8.3% next-generation BTKi’s, Fig. 2).

High (≥10%) AF burden was more common in those treated with ibrutinib compared to next-generation therapies (Supplemental Table 6). Yet, among those with AF, there was no significant difference in mean percent AF burden (Table 2, Supplemental Table 7). A greater proportion of next-generation BTKi users had VAs identified on rhythm monitors (29.2% vs 11.7%, *P* = 0.052). There were no statistically significant differences in the rates of other arrhythmias. Symptomatic or >1% PVCs were seen in 26.3% of next-generation BTKi users and 13.3% of ibrutinib users. Among next-generation BTKi users, rhythm monitors identified non-AF SVTs in 31.6%, symptomatic or >1% PACs in 13.2%, and both atrial and VA in 16.7%, respectively. In comparison, among ibrutinib users, monitors revealed other SVTs in 31.7%, symptomatic or >1% burden PACs in 10.0%, and concurrent atrial and VA in 6.7%, respectively. One patient on ibrutinib developed complete heart block; no sudden death was observed, inclusive of a median follow-up of 20 months in those with detected ventricular arrhythmias (Fig. 1). Identified arrhythmia led to cessation of BTKi therapy more frequently with ibrutinib therapy (31.7%) than next-generation therapy (10.5%, *P* = 0.016).

**Table 2:** Rhythm monitor results.

Variable, *n* (%)^a^	Next-generation BTKi (*n* = 38)	Ibrutinib (*n* = 60)	*P-*value
Monitor duration (days), mean (SD)	10.4 (11.4)	13.1 (13.4)	0.33
Long-term monitoring (>48 h), *n* (%)	17 (44.7)	28 (46.7)	1.0
Any Arrhythmia	28 (73.7)	43 (71.7)	0.83
AF	5 (13.2)	18 (30.0)	0.055
Incident AF	2 (6.9) (*n* = 29)	14 (25.5) (*n* = 55)	0.039
AF burden, mean (SD)	61.2% (47.1) (*n* = 5)	33.3% (41.6) (*n* = 18)	0.47
AF burden, median (SD)	99.0% (47.1)	10.0% (41.6)	0.65
High AF burden (≥10%)	3 (7.9)	11 (18.3)	0.35
Moderate AF burden (5–9.9%)	1 (2.6)	1 (1.7)	
Low AF burden (<5%)	34 (89.5)	48 (80.0)	
Ventricular arrhythmia (excluding PVCs)	7 (29.2))^b^	7 (11.7)	0.052
Ventricular arrhythmia (including symptomatic or >1% PVCs)	8 (33.3)^b^	15 (25.0)	0.27
PVCs (symptomatic or >1%)	10 (26.3)	10 (16.7)	0.58
PVC Burden (%), mean (SD)	7.1% (9.2) (*n* = 10)	9.4% (7.4) (*n* = 8)	0.57
Non-AF SVTs (excluding PACs)	12 (31.6)	19 (31.7)	0.99
Non-AF SVTs (including symptomatic or >1% PACs)	16 (42.1)	24 (40.0)	0.84
PACs (symptomatic or >1%)	5 (13.2)	6 (10.0)	0.63
Both atrial and ventricular arrhythmias (excluding PAC’s and PVC’s)	4 (16.7)^b^	4 (6.7)	0.16
Longest duration SVT (beats), mean (SD)	13.6 (17.2) (*n* = 10)	9.6 (7.9) (*n* = 16)	0.43
Longest duration NSVT, mean (SD)	6.7 (4.3) (*n* = 12)	5.2 (3.0) (*n* = 6)	0.46
2nd degree heart block	0 (0.0)	0 (0.0)	–
3rd degree (complete) heart block	0 (0.0)	1 (1.7)	0.42
Pauses	0 (0.0)	3 (5.0)	0.16
Sudden cardiac death	0 (0.0)	0 (0.0)	–
BTKi held/stopped due to arrhythmia	4 (10.5)	19 (31.7)	0.016
Anti-arrhythmic started	7 (18.4)	28 (46.7)	0.004
AF Ablation	2 (5.3)	5 (8.3)	0.57
VT Ablation	0 (0.0)	0 (0.0)	–
BTKi re-challenged	1 (2.6)	1 (1.7)	0.74

*AF* atrial fibrillation, *BTKi* Brutons tyrosine kinase inhibitor, *PACs* premature atrial contractions, *PVCs* premature ventricular contractions, *NSVT* non-sustained ventricular tachycardia, *SVT* supraventricular tachycardia, *VT* ventricular tachycardia.

^a^Except when otherwise specified.

^b^Denominators below listed means denote variables where a different number at risk is considered (e.g., denominator reflective of those without prior AF, in incident AF assessments).

### Effect of standard antiarrhythmic strategies

Among those patients in whom a single antiarrhythmic therapy was initiated or added and where multiple rhythm monitors were available, there was no difference in subsequent control of AF burden across time (Supplemental Table 6A); there was a nonsignificant trend toward reduction in those managed with the addition of ≥2 antiarrhythmics. Similarly, in those with precedent antiarrhythmic use, no class was associated with the prevention of subsequent high AF burden development. However, the initiation of any new antiarrhythmic or procedure therapy was associated with a reduction in overall arrhythmic burden (−35.8% vs. 0% reduction, *P* = 0.009; Supplemental Table 6B).

### Effect of AF burden on future major cardiovascular events and mortality

Over a median follow-up of 18 months (IQR 10–34 months; range 1 week–93 months), 35.7% developed MACE, including 71.4% of those with high AF burden after BTKi initiation. AF-related hospitalization was the most frequent subsequent event (25.6%), followed by CHF (10.2%), transient ischemic attack (6.1%), and stroke (6.1%) development; Supplemental Table 8). In univariate analysis, high AF burden was associated with subsequent MACE (HR 2.95, *P* = 0.004, Supplemental Table 9). Similarly, high AF burden is associated with increased all-cause mortality (HR 3.18, *P* = 0.003, Supplemental Table 10).

**Fig. 3:**
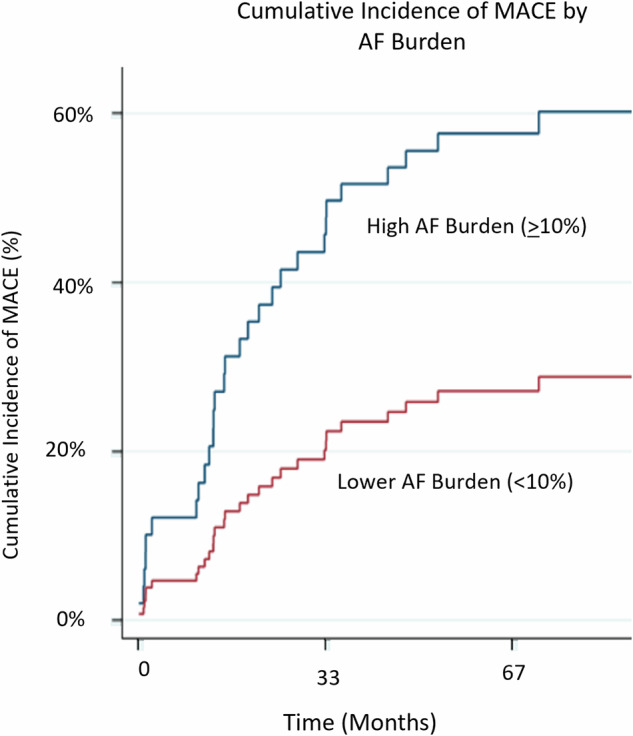
Major cardiovascular events during BTKi treatment, by degree of AF burden following BTKi treatment initiation. High AF burden defined as ≥10% of all beats over 24 h being AF; with drug discontinuation and non-cardiac mortality treated as competing risks. AF atrial fibrillation; BTKi Bruton’s tyrosine kinase inhibitor; MACE major cardiovascular events.

**Fig. 4:**
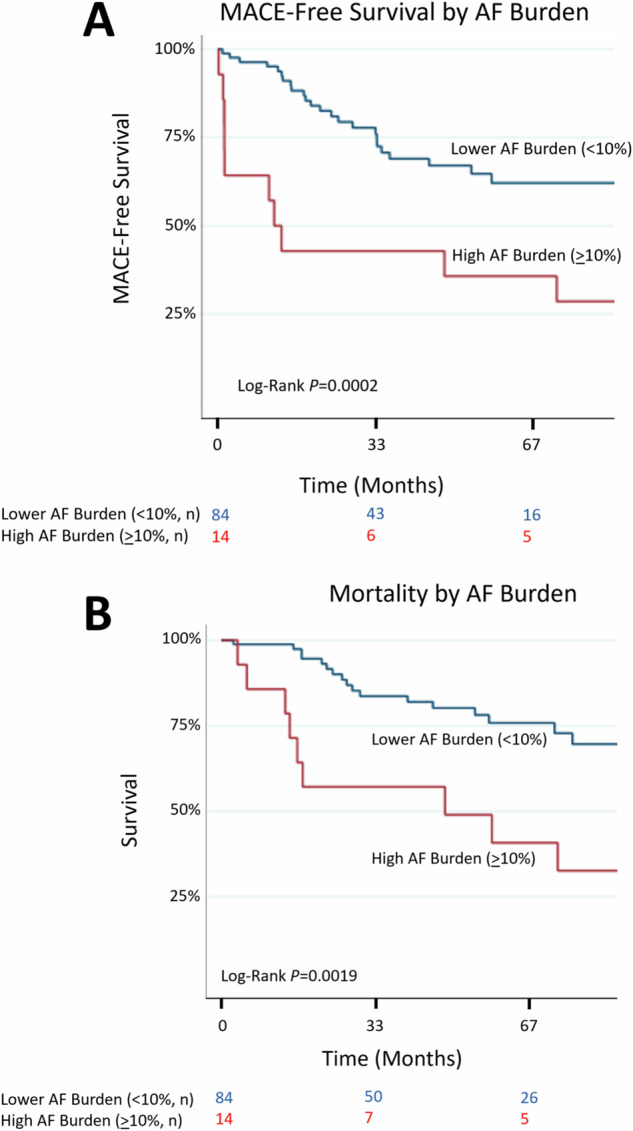
MACE-free survival, and overall survival by degree of AF burden following BTKi treatment initiation. **A** High AF burden defined as ≥ 10% of all beats over 24 h being AF, associates with worse MACE-free survival and (**B**) all-cause mortality. AF atrial fibrillation; BTKi Bruton’s tyrosine kinase inhibitor; MACE major cardiovascular events.

Moreover, in multivariable models accounting for traditional cardiac risk factors, high AF burden remained associated with increased MACE (HR 3.12, *P* = 0.005) and mortality (HR 3.0, *P* = 0.007) in longer-term follow-up; Figs. 3 and 4; Table 3; Supplemental Tables 11 and 12). In landmark analysis, excluding AF events <30 days from cardiac rhythm monitoring initiation, AF burden remained associated with increased MACE (Supplemental Fig. 2, Supplemental Tables 9–11). ROC analysis revealed similar relation between higher AF burden, and subsequent MACE or death (ROC 0.7, *P* < 0.05; Supplemental Fig. 3).

**Table 3:** Multivariable predictors of all-cause mortality, by AF burden.

Variable	Hazards ratio	95% Confidence interval	*P*-value
High AF burden (≥10%)	2.97	1.35–6.54	0.007
Prior AF	1.52	0.54–4.32	0.43
Primary malignancy CLL	0.56	0.23–1.40	0.22
Total duration of (any) BTKi therapy^a^, months	1.01	0.998–1.03	0.09

*AF* atrial fibrillation, *BTKi* Bruton’s tyrosine kinase inhibitor, *CLL* chronic lymphocytic leukemia.

^a^At time of monitoring.

## Discussion

In this evaluation of the burden of AF among patients who underwent extended ECG monitoring for suspected BTKi-associated cardiotoxicity, nearly three-quarters had detectable arrhythmias including nearly 20% with high burdens of AF or other serious arrhythmias. Among those with detectable arrhythmias, the median burden of AF was 10%. The likelihood of higher AF burden was markedly increased in those with a history of AF prior to BTKi therapy, and greatest in those taking ibrutinib. Yet, we also noted that initiation of an antiarrhythmic therapy or procedure after cardiotoxicity development was associated with reduction in subsequent overall arrhythmic burden. Those patients with high AF burden also saw a 3-fold increase in subsequent MACE and mortality. This pattern remained even after accounting for the duration of BTKi treatment and traditional CVD risk factor burden. These observations are especially relevant given the relative lack of trackable markers to interpret the future risk of serious events or death in those with suspected cardiotoxicity.

The observation of increased AF burden in patients treated with BTKi’s adds to a growing body of evidence linking BTKi with potential cardiac toxicity. Yet, to date, a limited number of studies have evaluated the exact burden or impacts of arrhythmia development following BTKi-initiation, particularly including next-generation therapies (e.g., acalabrutinib, pirtobrutinib). To our knowledge, this study represents the largest systematic investigation of the nature and impacts of AF, VA, and other arrhythmia development on subsequent MACE and overall survival; and the first evaluation of the burden of arrhythmias with next-generation BTKi-therapy. In a study focused on incident AF development following ibrutinib initiation, over 30% of CLL patients developed AF following treatment initiation with systematic cardiac rhythm evaluation [22]. Similarly, in an investigation of 72 ibrutinib-treated patients with suspected cardiotoxicity, nearly 87% developed a detectable arrhythmia, including nearly 26% with incident AF and over 40% with NSVT or arrhythmia events [15]. Yet, the effect of next-generation BTKi’s on arrhythmia burden was not assessed. Within the current study, we observed no difference in the burden of AF and other arrhythmias with next-generation therapies in those with suspected cardiotoxicity.

The predictors of MACE following BTKi therapy initiation are not well understood. Data from prior studies have suggested that traditional factors, including age and hypertension development or worsening, may underlie, at least in part, a significant proportion of the observed risk [23]. However, many of these events are not well explained by traditional factors alone, with many patients having almost no risk factors outside of BTKi-treatment [6, 9]. Although the presence of AF itself did not remain associated with mortality across multiple cardiac and cancer strata or factors, the presence of high burdens of AF strongly predicted subsequent MACE and mortality. This may lend credence to the consideration of the frequency or burden of AF (and other arrhythmias) in the assessment of subsequent mortality risk, particularly among patients with suspected cardiotoxic symptoms. Akin to patients with significant cardiomyopathy, high AF burden may serve as a key marker for understanding the cardiotoxic effects of BTKi’s on survival outcomes. While ventricular arrhythmic events may result in more overt or sudden mortality, increased AF burden more insidiously associated with stroke and mortality. In the current study, high AF burden and other arrhythmias led to increase in MACE not characterized in earlier more anticancer efficacy-focused studies [1, 3–5, 24–26].

In the current study, higher VA burden did not clearly link with higher MACE or mortality risk. Given the potential high anticancer efficacy of most BTKi’s, it may be reasonable to suggest that these therapies generally not be held for arrhythmic reasons in the absence of sustained or prolonged VAs, arrhythmic symptom intolerance, or other high-risk event (e.g., sudden death). Yet, given the potential consequences of VAs, larger prospective cardiac studies are needed to inform the optimal management strategy in those with higher VA burden.

The effects of cardiotoxicity following BTKi therapy on survival outcomes are not well defined. Beyond traditional cancer risk factors, high AF burden associated with worse MACE-free and overall survival. While the observed relationships between higher AF and MACE were similar to those seen in non-cancer populations, the median burden of AF with BTKi’s was higher than the 2–8% reported with more traditional AF [27–32]. Among cancer patients, higher AF burden is a potent driver of treatment intolerance and discontinuation. Since worse outcomes were observed in those with high AF burden, it stands to reason that minimizing time in AF may potentially serve a treatment goal, especially for the continuation of BTKi therapy to reap maximum efficacy [33]. Yet, in conjunction with the observation that no specific antiarrhythmic intervention appeared to prevent or definitively reduce AF burden, additional studies are needed to confirm the optimal strategy for AF control with cancer therapies.

Although the exact mechanisms behind these observations are not clear, animal models and available myocardial biopsy studies demonstrate chronic BTKi’s lead to myocardial remodeling, with increased inflammation, fibrosis, and subsequent chamber dilation [34]. Notably, in non-BTKi models, higher AF burden serves as a key driver of pro-fibrotic remodeling and arrhythmia perpetuation [35–37]. With ibrutinib and other BTKi’s, these processes may be generally exaggerated, which may offer a key substrate for serious cardiotoxic sequelae. As prior investigations have focused on the incidence of BTKi-induced AF, systematic longer-term comprehensive cardiac monitoring studies, inclusive of AF and VT burden, have been largely unavailable. In our cohort, nearly 11% of patients with arrhythmias (e.g., AF, VAs) ultimately saw treatment held or stopped entirely. This decision is often driven by the burden or intensity of toxicity symptoms, which is increased among those with higher AF burden. In conjunction with potential unexplained deaths in those with VAs [9], these effects may be a key contributor to the increased mortality among those with high AF burdens. This is supported by anticancer efficacy-focused studies of BTKi treatment alteration or interruptions. However, additional prospective studies are needed to elucidate the exact nature and pathways of these events.

### Limitations

Several limitations should be acknowledged. Rhythm monitor data were primarily available in those with suspected cardiotoxicity, as opposed to asymptomatic patients. Given the retrospective nature of this study, available follow-up and duration of monitoring were not uniform. Despite accounting for variation in follow-up and traditional risk factors via extensive modeling, observed rates from comparator oncologic populations were not routinely available. Compliance to potential AF preventative therapies could not be determined. The decision to initiate antiarrhythmic therapy was at the discretion of the treating clinicians and reflected routine practice patterns. Similarly, the selected class or strategy and dose of therapy were not predetermined. Some groups have reported the longest AF episode or frequency of AF episodes based on how individual monitor devices produce reports in non-cancer patients, but we elected to report AF burden as percentage of time spent in AF during the monitoring period, in line with published consensus definitions [18]. We could not clearly determine the effect of specific BTKi switches on AF or VA burden. The majority of next-generation BTKi therapy was with acalabrutinib; as other BTKi’s (e.g., zanubrutinib, pirtobrutinib) were not yet approved. Beyond AF detection, rhythm monitor reports were not uniform, as different types and brands were used. While wearable ambulatory rhythm monitors capture more than 10 s ECGs, they still did not capture long durations (e.g., 2 years) of cardiac rhythms as with implanted monitoring. Finally, some out-of-hospital cardiac events may have gone uncaptured, despite extensive search.

In summary, BTKi’s associated with risk of substantial burden of arrhythmic events, even after accounting for traditional risk factors. Beyond traditional risk, high AF burden in those suspected of BTKi-cardiotoxicity associated with increased risk of major cardiovascular events and worse survival outcomes, even among those receiving next-generation therapies. Given the anticipated increase in patients receiving BTKi’s, additional studies focused on the relationship of AF burden to subsequent outcomes and optimal mitigation strategies are needed.

## Supplementary information

Supplemental Materials - Clean

## Data Availability

Original data available upon reasonable request; please contact daniel.addison@osumc.edu.
