# Supplementary material for: Atrial fibrillation burden and clinical outcomes following BTK inhibitor initiation
Source: Leukemia. 2024 Aug 17;38(10):2141–9. doi: 10.1038/s41375-024-02334-3 (PMC11436370; doi:10.1038/s41375-024-02334-3)
Supplement: Supplementary file 1 — Supplemental Materials - Clean [file 41375_2024_2334_MOESM1_ESM.docx]

**Supplemental Material:**

**sTable 1.** Baseline Characteristics, stratifying next-generation BTKi therapy by prior ibrutinib use.

| **Characteristic** | **Total**  **(n=98)** | **Next-Generation BTKi (Ibrutinib naïve, n=24)** | **Next-Generation BTKi (prior Ibrutinib, n=14)** | **Ibrutinib (n=60)** | ***P*-value** |
| --- | --- | --- | --- | --- | --- |
| BTK Inhibitor, n (%) | | | | | |
| Acalabrutinib | 34 (34.7) | 24 (100) | 10 (71.4) | - | - |
| Ibrutinib | 60 (61.2) | - | - | 60 (100) |  |
| Nemtabrutinib (ARQ-531) | 3 (3.1) | 0 (0.0) | 3 (21.4) | - |  |
| Pirtobrutinib (LOXO 305) | 1 (1.0) | 0 (0.0) | 1 (7.1) | - |  |
| Age (years) | 68.5 (8.9) | 66.3 (9.1) | 70.1 (7.1) | 69.0 (9.2) | 0.35 |
| Sex, n (%) | | | | | |
| Male | 71 (72.4) | 19 (79.2) | 10 (71.4) | 42 (70.0) | 0.69 |
| Female | 27 (27.6) | 5 (20.8) | 4 (28.6) | 18 (30.0) |  |
| BMI (kg/ m^2^), mean (SD) | 28.5 (6.3) | 28.1 (6.4) | 29.0 (5.0) | 28.56 (6.59) | 0.92 |
| Other baseline traditional AF risk factors, n (%) | | | | | |
| CHF | 3 (3.1) | 1 (4.2) | 0 (0.0) | 2 (3.3) | 0.76 |
| Valvular Disease | 4 (4.1) | 1 (4.2) | 0 (0.0) | 3 (5.0) | 0.70 |
| Hypertension | 52 (53.1) | 15 (62.5) | 9 (64.3) | 28 (46.7) | 0.28 |
| Hyperlipidemia | 34 (34.7) | 12 (50.0) | 3 (21.4) | 19 (31.7) | 0.15 |
| DM | 18 (18.4) | 1 (4.2) | 4 (28.6) | 13 (21.7) | 0.10 |
| MI | 7 (7.1) | 1 (4.2) | 1 (7.1) | 5 (8.3) | 0.80 |
| OSA | 17 (17.3) | 5 (20.8) | 2 (14.3) | 10 (16.7) | 0.86 |
| CKD | 18 (18.4) | 2 (8.3) | 1 (7.1) | 15 (25.0) | 0.10 |
| Any Prior Arrhythmia | 27 (27.6) | 7 (29.2) | 6 (42.9) | 14 (23.3) | 0.33 |
| Prior AF | 14 (14.3) | 5 (20.8) | 4 (28.6) | 5 (8.3) | 0.11 |
| Prior SVT | 8 (8.2) | 2 (8.3) | 0 (0.0) | 6 (10.0) | 0.47 |
| Prior VA | 4 (4.1) | 1 (4.2) | 1 (7.1) | 2 (3.3) | 0.81 |
| Prior BTKi-associated arrhythmia | 5 (5.1) | 1 (4.2) | 4 (28.6) | 0 (0.0) | <0.001 |
| Smoking Status, n (%) | | | | | |
| Never | 56 (57.1) | 14 (58.3) | 6 (42.9) | 36 (60.0) | 0.50 |
| Former (or current) | 42 (42.9) | 10 (41.7) | 8 (57.1) | 24 (40.0) |  |
| Primary malignancy, n (%) | | | | | |
| CLL | 86 (87.8) | 23 (95.8) | 14 (100) | 49 (81.7) | 0.07 |
| MCL | 6 (6.1) | 0 (0.0) | 0 (0.0) | 6 (10.0) |  |
| Other* | 6 (6.1) | 1 (4.2) | 0 (0.0) | 5 (8.3) |  |
| Rai stage, mean (SD) | 2.7 (1.4)  (n=79) | 2.3 (1.4)  (n=24) | 2.1 (1.5)  (n=11) | 3.02 (1.32)  (n=44) | 0.036 |
| Baseline ECOG performance status, mean (SD) | 0.5 (0.6) | 0.63 (0.58) | 0.57 (0.51) | 0.47 (0.59) | 0.54 |
| Number of prior anticancer therapies, median (IQR) | 2 (1-3) | 0 (0-2) | 3 (1-3) | 2 (1-3) | <0.001 |
| Concomitant Chemotherapy, n (%) | 30 (30.7) | 11 (45.8) | 1 (7.1) | 18 (30.0) | 0.044 |
| Prior Chemotherapy, n (%) | 59 (60.2) | 7 (29.2) | 10 (71.4) | 42 (70.0) | 0.002 |
| Prior Monoclonal Antibody, n (%) | 70 (71.4) | 8 (33.3) | 12 (85.7) | 50 (83.3) | <0.001 |
| Prior BTKi Therapy, n (%) | 16 (16.3) | 0 (0.0) | 14 (100) | 2 (3.3) | <0.001 |
| Prior Immunomodulatory therapy, n (%) | 14 (14.3) | 3 (12.5) | 2 (14.3) | 9 (15.0) | 0.96 |
| No prior anticancer therapies, n (%) | 4 (4.1) | 0 (0.0) | 0 (0.0) | 4 (6.7) | 0.27 |
| Total Duration of (any) BTKi Therapy at time of rhythm monitor, months, mean (SD) | 42.0 (28.7) | 44.9 (28.8) | 59.8 (28.8) | 36.8 (27.4) | 0.96 |
| Duration of “Current” BTKi Therapy at time of rhythm monitor, months, mean (SD) | 37.0 (27.2) | 44.9 (28.8) | 26.5 (19.7) | 36.3 (27.5) | 0.31 |
| LA Volume Index ml/m^2^ , mean (SD) | 19.4 (12.5)  (n=14) | 22.4 (9.1)  (n=6) | 23.4 (16.5)  (n=2) | 15.0 (10.7)  (n=6) | 0.89 |
| LVEF (%) | 60.6 (5.6)  (n=49) | 62.8 (3.8)  (n=20) | 59.0 (4.2)  (n=5) | 59.2 (6.7)  (n=24) | 0.041 |
| Baseline Cardiac Medications | | | | | |
| Beta-blocker | 20 (20.4) | 4 (16.7) | 5 (35.7) | 11 (18.3) | 0.31 |
| Calcium channel blocker | 13 (13.3) | 5 (20.8) | 2 (14.3) | 6 (10.0) | 0.41 |
| Amiodarone | 1 (1.0) | 0 (0.0) | 0 (0.0) | 1 (1.7) | 0.73 |
| Other anti-arrhythmic | 1 (1.0) | 0 (0.0) | 0 (0.0) | 1 (1.7) | 0.73 |
| Prior AF Ablation | 5 (5.1) | 1 (4.2) | 2 (14.3) | 2 (3.3) | 0.24 |
| Care Team | | | | | |
| Cardio-Oncologist | 20 (20.4) | 7 (29.2) | 3 (21.4) | 10 (16.7) | 0.44 |
| Electrophysiologist | 28 (28.6) | 7 (29.2) | 2 (14.3) | 19 (31.7) | 0.43 |
| Any cardiologist | 89 (90.1) | 18 (75.0) | 13 (92.9) | 58 (96.7) | 0.008 |

Abbreviations: AF, atrial fibrillation; BMI, body mass index; BTKi, Brutons tyrosine kinase inhibitor; CLL, chronic lymphocytic leukemia; CHF, congestive heart failure; CKD, chronic kidney disease; DM, diabetes mellitus; ECOG, eastern cooperative group; IQR, interquartile range; LA, left atrial; LVEF, left ventricular ejection fraction; MCL, mantle cell lymphoma; MI, myocardial infarction; OSA, obstructive sleep apnea; SD, standard deviation; SVT, supraventricular tachycardia; VA, ventricular arrhythmia. * Diffuse large B-cell lymphoma, follicular lymphoma, hairy cell leukemia, and marginal zone lymphoma.

**sTable 2.** Baseline Characteristics, by atrial fibrillation (AF) burden status. *High AF burden defined as ≥10% of beats over a 24-hour period being AF.

| **Characteristic** | **High AF Burden (n=14)** | **Low AF Burden (n=84)** | ***P* value** |
| --- | --- | --- | --- |
| Age at monitor initiation, mean (SD) | 69.8 (6.2) | 68.2 (9.3) | 0.54 |
| Sex, n (%) | | | |
| Male | 12 (86) | 59 (70) | 0.23 |
| Female | 2 (14) | 25 (30) |  |
| BTKi therapy | | | |
| Ibrutinib | 11 (79) | 49 (58) | 0.15 |
| Next- Generation BTKi* | 3 (21) | 35 (42) |  |
| BMI (kg/m^2^), mean (SD) | 30.0 (8.6) | 28.3 (5.9) | 0.36 |
| Other baseline traditional risk factors | | | |
| CHF | 1 (7.1) | 2 (2.4) | 0.34 |
| Valvular Disease | 1 (7.1) | 4 (4.8) | 0.71 |
| Hypertension | 7 (50) | 45 (54) | 0.80 |
| Hyperlipidemia | 4 (29) | 30 (36) | 0.60 |
| DM | 0 (0) | 18 (21) | 0.06 |
| MI | 1 (7.1) | 1 (7.1) | 0.99 |
| OSA | 1 (7.1) | 16 (19) | 0.28 |
| CKD | 5 (36) | 13 (16) | 0.07 |
| Prior AF | 4 (29) | 8 (9.5) | 0.044 |
| Smoking Status | | | |
| Never | 7 (50) | 49 (58) | 0.56 |
| Former (or current) | 7 (50) | 35 (42) |  |
| Primary malignancy, n (%) | | | |
| CLL | 10 (71) | 76 (90) | 0.044 |
| Mantle Cell Lymphoma | 4 (29) | 8 (10) |  |
| Other** | 4 (29) | 8 (10) |  |
| Rai stage, mean (SD) | 3.2 (1.4) | 2.6 (1.4) | 0.21 |
| Baseline ECOG performance status, mean (SD) | 0.29 (0.61) | 0.57 (0.57) | 0.09 |
| Treatment History, n (%) | | | |
| Number of prior anticancer therapies, median (IQR) | 2 (1–3) | 2 (1–3) | 0.62 |
| Concomitant Chemotherapy | 4 (29) | 26 (31) | 0.86 |
| Prior Chemotherapy | 8 (57) | 51 (61) | 0.80 |
| Prior Monoclonal Antibody | 11 (79) | 59 (70) | 0.52 |
| Prior BTKi Therapy | 1 (7.1) | 15 (18) | 0.32 |
| Prior Immunomodulatory therapy | 2 (14) | 12 (14) | 0.99 |
| No prior anticancer therapies, n (%) | 1 (7.1) | 3 (3.6) | 0.53 |
| Duration of prior BTKi treatment | | |  |
| Total Duration of (any) BTKi therapy at  time of rhythm monitor, months, mean (SD) | 28 (26) | 39 (27) | 0.17 |
| Duration of Ibrutinib therapy at time of  rhythm monitor, months, mean (SD) | 21 (24) | 24 (28) | 0.64 |
| Duration of Next-Gen therapy at time of  rhythm monitor, months, mean (SD) | 7.4 (20) | 20 (30) | 0.13 |
| LA Volume Index (ml/m^2^) | 11.5 (5.4) | 22.5 (13.9) | 0.16 |
| LVEF (SD) | 57% (9%) | 61 (5%) | 0.065 |
| Baseline anti-arrhythmic and rate control medications | | |  |
| Beta-blocker | 5 (36) | 15 (18) | 0.13 |
| Calcium channel blocker | 2 (14) | 11 (13) | 0.90 |
| Amiodarone | 0 (0) | 1 (1.2) | 0.68 |
| Other anti-arrhythmic | 0 (0) | 1 (1.2) | 0.68 |
| Prior AF Ablation | 3 (21) | 2 (2.4) | 0.003 |
| Rhythm Monitor Duration (days), mean (SD) | 7.3 (12.3) | 12.8 (12.9) | 0.14 |
| Specialty Provider evaluation | | | |
| Cardio-Oncologist | 2 (14) | 18 (21) | 0.54 |
| Electrophysiologist | 8 (57) | 20 (24) | 0.011 |
| Any cardiologist | 14 (100) | 75 (89) | 0.20 |

Abbreviations: AF, atrial fibrillation; BMI, body mass index; BTKi, Bruton's Tyrosine Kinase inhibitor; CHF, congestive heart failure; CKD, chronic kidney disease; CLL, chronic lymphocytic leukemia; DM, diabetes mellitus; ECOG, Eastern Cooperative Oncology Group; IQR, interquartile range; LA, left atrium; LVEF, left ventricular ejection fraction; MI, myocardial infarction; OSA, obstructive sleep apnea; SD, standard deviation . *Next-gen BTKi includes acalabrutinib and non-covalent drugs (here pirtobrutinib, nemtabrutinib). **Diffuse large B-cell lymphoma, follicular lymphoma, hairy cell leukemia, and marginal zone lymphoma

**sTable 3.** Rhythm Monitor Indications, generation of BTKi.

| **Variable** | **Next Generation BTKi (n=38)** | **Ibrutinib**  **(n=60)** | ***P-*value** |
| --- | --- | --- | --- |
| **Rhythm Monitor Indication** | | | |
| Palpitations | 16 (42.1) | 23 (38.3) | 0.71 |
| Dyspnea on Exertion | 1 (2.7) | 0 (0.0) | 0.21 |
| Fatigue | 1 (2.7) | 0 (0.0) | 0.21 |
| AF (old or new) | 12 (31.6) | 23 (38.3) | 0.50 |
| Syncope/Pre-Syncope | 4 (10.5) | 5 (8.3) | 0.71 |
| TIA/Stroke | 0 (0.0) | 2 (3.3) | 0.26 |
| Chest Pain | 0 (0.0) | 4 (6.7) | 0.10 |
| Abnormal ECG or other (non-AF) arrhythmia | 9 (23.7) | 11 (18.3) | 0.52 |
| Required by Clinical Trial (or asymptomatic) | 0 (0.0) | 0 (0.0) | 0.71 |

Abbreviations: AF, atrial fibrillation; BTKi, Bruton’s tyrosine kinase inhibitor; ECG, electrocardiogram; TIA, transient ischemic attack.

**sTable 4.** Univariate predictors of high BTKi-related AF burden.

| **Variable** | **Hazards Ratio** | **95% Confidence Interval** | ***P*-value** |
| --- | --- | --- | --- |
| Prior AF | 3.8 | 0.97 – 14.9 | 0.056 |
| DM | 1 | - | - |
| Prior AF Ablation | 11.2 | 1.68 – 74.5 | 0.013 |
| Total Duration of (any) BTKi Therapy at time of rhythm monitor, months | 0.98 | 0.95 – 1.00 | 0.06 |
| Total Duration of ibrutinib therapy at time of rhythm monitor, months | 0.99 | 0.97-1.02 | 0.63 |
| Total Duration of next-generation BTKi Therapy at time of rhythm monitor, months | 0.98 | 0.95-1.01 | 0.15 |
| Primary Malignancy of CLL | 0.26 | 0.067 – 1.03 | 0.06 |
| LVEF | 0.89 | 0.78 – 1.02 | 0.08 |
| LVEF Low (≤ 50%) | 3.15 | 0.27 – 37.3 | 0.36 |
| HTN | 0.87 | 0.28 – 2.69 | 0.80 |
| Total Duration of (any) BTKi Therapy less than 1 year | 6.83 | 0.88 – 53.2 | 0.07 |
| Next Generation BTKi* | 0.38 | 0.10 – 1.47 | 0.16 |

Abbreviations: AF, atrial fibrillation; BTKi, Bruton's Tyrosine Kinase inhibitor; CLL, chronic lymphocytic leukemia; DM, diabetes mellitus; HTN, hypertension; LVEF, left ventricular ejection fraction; *Next-generation BTKi includes acalabrutinib and non-covalent drugs (here pirtobrutinib, nemtabrutinib). **Logistic regression model for high AF Burden (≥10%) with univariate for p< 0.05.

**sTable 5A.** Multivariable analyses for predictors of high (>10%) atrial fibrillation burden.

| **Variable** | **Hazards Ratio** | **95% Confidence Interval** | ***P*-value** |
| --- | --- | --- | --- |
| CLL | 0.22 | 0.05 – 0.93 | 0.04 |
| Prior AF | 4.47 | 1.07 – 18.6 | 0.04 |

Abbreviations: AF, atrial fibrillation; CLL, chronic lymphocytic leukemia

**sTable 5B.** Multivariable analyses for predictors of high (>10%) atrial fibrillation burden.

| **Variable** | **Hazards Ratio** | **95% Confidence Interval** | ***P*-value** |
| --- | --- | --- | --- |
| Next-Generation BTKi* | 0.23 | 0.05 – 1.08 | 0.06 |
| Prior AF | 6.73 | 1.36 -33.2 | 0.019 |

Abbreviations: AF, atrial fibrillation; BTKi, Bruton’s Tyrosine Kinase inhibitor *Next-generation BTKi includes acalabrutinib and non-covalent drugs (here pirtobrutinib, nemtabrutinib)

**sTable 6A.** Prevention of incident AF during BTKi therapy among BTKi users, by anti-arrhythmic therapy (n=19).*

| **Treatment** | **Hazard Ratio** | **95% CI (lower-upper)** | ***P*-value** |
| --- | --- | --- | --- |
| Beta Blocker† (n=9) | 1.34 | 0.3-5.98 | 0.70 |
| Calcium Channel Blocker (n=6) | 0.88 | 0.11-6.77 | 0.90 |
| Combination‡ (n=4) | 1.28 | 0.17-9.91 | 0.81 |
| Other (n=0) | - | - | - |

Abbreviations: AF, atrial fibrillation; BTKi, Bruton’s tyrosine kinase inhibitor; CI, confidence interval; *No patients were on baseline sodium channels. †Includes metoprolol, carvedilol, and sotalol. ‡Subjects on ≥ 2 antiarrhythmics

**sTable 6B.** Change in arrhythmia burden during BTKi therapy, among subjects requiring the addition of only a single new or additional antiarrhythmic medication or procedure following initial rhythm monitor detected BTKi arrhythmia. (From the 17 patients with multiple available extended ECG monitor measures, 5 were started on a new or additional antiarrhythmic therapy for arrhythmia control and had pre and post intervention burden measures during BTKi use).

| **Treatment** | **No. with therapy added (n = 5, %)** | **Change in AF burden, % (Post - Pre), mean** | **Change in PVC burden*****, % (Post - Pre), mean** |
| --- | --- | --- | --- |
| Beta Blocker† | 3 (60.0) | -66.7 | -10.2 |
| Calcium Channel Blocker | 1 (20.0) | +26 | 0 |
| Sodium Channel Blocker | 1 (20.0) | +26 | 0 |
| Cardioversion | 2 (40.0) | -37 | 0 |
| Ablation | 2 (40.0) | -52 | 0 |
| Other | 0 | n/a | n/a |
| Combination‡ | 3 | -58 (59.4) | 0 |
| Overall | 5 (100) | -35.8 (53.5) | -6.2 (12.4) |

Abbreviations: AF, atrial fibrillation; BTKi, Bruton’s tyrosine kinase inhibitor; ECG, electrocardiogram; PVC, premature ventricular contraction; VT, ventricular tachycardia. *Include runs of non-sustained VT. †Includes metoprolol and sotalol. ‡Subjects requiring ≥ 2 antiarrhythmics and/or procedural intervention to be initiated during BTKi use.

**sTable 7A.** Rhythm Monitor Results, acalabrutinib vs. ibrutinib alone.

| **Variable, n (%)*** | **Acalabrutinib (n=34)** | **Ibrutinib**  **(n=60)** | ***P-*value** |
| --- | --- | --- | --- |
| Monitor Duration (days), mean (SD) | 10.9 (12.0) | 13.1 (13.4) | 0.47 |
| Long-term monitoring (>48 hours), n (%) | 15 (44.1) | 28 (46.7) | 0.057 |
| Any Arrhythmia | 25 (73.6) | 43 (71.7) | 0.85 |
| AF | 5 (14.7) | 18 (30.0) | 0.10 |
| Incident AF | 2 (8.0)  (n=25) | 14 (25.5)  (n=55) | 0.07 |
| AF Burden, mean (SD) | 61.2% (47.1)  (n=5) | 33.3% (41.6)  (n=18) | 0.21 |
| AF Burden, median (IQR) | 99.0% (45-100) | 10.0% (4-100) | 0.68 |
| High AF Burden (>10%) | 3 (8.8) | 11 (18.3) | 0.44 |
| Moderate AF Burden (5-9.9%) | 1 (2.9) | 1 (1.7) |  |
| Low AF Burden (<5%) | 30 (88.2) | 48 (80.0) |  |
| Ventricular Arrhythmia (excluding PVCs) | 7 (29.2)** | 7 (11.7) | .029 |
| Ventricular Arrhythmia (including symptomatic or >1% PVCs) | 8 (33.3)** | 15 (25.0) | 0.44 |
| PVCs (symptomatic or >1%) | 8 (23.5) | 10 (16.7) | 0.42 |
| PVC Burden (%), mean (SD) | 7.9% (10.0)  (n=8) | 9.4% (7.4)  (n=8) | 0.74 |
| Non-AF SVTs (excluding PACs) | 11 (32.4) | 19 (31.7) | 0.95 |
| Non-AF SVTs (including symptomatic or >1% PACs) | 14 (41.2) | 24 (40.0) | 0.91 |
| PACs (symptomatic or >1%) | 4 (11.8) | 6 (10.0) | 0.79 |
| Both atrial and ventricular arrhythmias (excluding PAC’s and PVC’s) | 4 (16.7)** | 4 (6.7) | 0.16 |
| Longest Duration SVT (beats), mean (SD) | 12.9 (17.2) (n=10) | 9.6 (7.9) (n=16) | 0.51 |
| Longest Duration NSVT, mean (SD) | 6.7 (4.3) (n=12) | 5.2 (3.0) (n=6) | 0.46 |
| 2^nd^ Degree Heart Block | 0 (0.0) | 0 (0.0) | - |
| 3^rd^ Degree (Complete) Heart Block | 0 (0.0) | 1 (1.7) | 0.45 |
| Pauses | 0 (0.0) | 3 (5.0) | 0.20 |
| Sudden Cardiac Death | 0 (0.0) | 0 (0.0) | - |
| BTKi held/stopped due to arrhythmia | 4 (11.8) | 19 (31.7) | 0.031 |
| Anti-arrhythmic started | 7 (20.6) | 28 (46.7) | 0.012 |
| AF Ablation | 2 (5.9) | 5 (8.3) | 0.66 |
| VT Ablation | 0 (0.0) | 0 (0.0) | - |
| BTKi re-challenged | 1 (2.9) | 1 (1.7) | 0.68 |

Abbreviations: AF, atrial fibrillation; BTKi, Brutons tyrosine kinase inhibitor; PACs, premature atrial contractions; PVCs, premature ventricular contractions; NSVT, non-sustained ventricular tachycardia; SVT, supraventricular tachycardia; VT, ventricular tachycardia. *Except when otherwise specified. **Excludes patients previously on ibrutinib

**sTable 7B.** Rhythm Monitor Results, with any next-generation BTKi therapy (by prior ibrutinib use status) vs. Ibrutinib.

| **Variable, n (%)*** | **Next-Generation BTKi (Ibrutinib naïve, n=24)** | **Next-Generation (prior Ibrutinib, n=14)** | **Ibrutinib**  **(n=60)** | ***P-*value** |
| --- | --- | --- | --- | --- |
| Monitor Duration (days), mean (SD) | 9.9 (12.7) | 11.0 (10.3) | 13.1 (13.4) | 0.60 |
| Long-term monitoring (>48 hours), n (%) | 9 (37.5) | 8 (57.1) | 28 (46.7) | 0.49 |
| Any Arrhythmia | 17 (70.8) | 11 (78.6) | 43 (71.7) | 0.86 |
| AF | 4 (16.7) | 1 (7.1) | 18 (30.0) | 0.13 |
| Incident AF | 2 (10.5)  (n=19)** | 0 (0.0) | 14 (25.5)  (n=55)** | 0.06 |
| AF Burden, mean (SD) | 51.8% (55.7)  (n=4)** | 99.0% (-)  (n=1)** | 33.3% (41.6)  (n=18)** | 0.55 |
| AF Burden, median (SD) | 52.5% (55.7) | - | 10.0% (41.6) | 0.81 |
| High AF Burden (>10%) | 2 (8.3) | 1 (7.1) | 11 (18.3) | 0.47 |
| Moderate AF Burden (5-9.9%) | 1 (4.2) | 0 (0.0) | 1 (1.7) |  |
| Low AF Burden (<5%) | 21 (87.5) | 13 (92.9) | 48 (80.0) |  |
| Ventricular Arrhythmia (excluding PVCs) | 7 (29.2)*** | 5 (35.7) | 7 (11.7) | 0.046 |
| Ventricular Arrhythmia (including symptomatic or >1% PVCs) | 8 (33.3)*** | 7 (50.0) | 15 (25.0) | 0.18 |
| PVCs (symptomatic or >1%) | 5 (20.8) | 5 (35.7) | 10 (16.7) | 0.28 |
| PVC Burden (%), mean (SD) | 10.1% (13.1)  (n=5)** | 4.15% (4.0)  (n=5)** | 9.4% (7.4)  (n=8)** | 0.51 |
| Non-AF SVTs (excluding PACs) | 9 (37.5) | 3 (21.4) | 19 (31.7) | 0.59 |
| Non-AF SVTs (including symptomatic or >1% PACs) | 9 (37.5) | 6 (42.9) | 24 (40.0) | 0.95 |
| PACs (symptomatic or >1%) | 2 (8.3) | 3 (21.4) | 6 (10.0) | 0.42 |
| Both atrial and ventricular arrhythmias (excluding PAC’s and PVC’s) | 4 (16.7)*** | 1 (7.1) | 4 (6.7) | 0.34 |
| Longest Duration SVT (beats), mean (SD) | 8.1 (5.9)  (n=8)** | 28.3 (30.2)  (n=3)** | 9.6 (7.9)  (n=17)** | 0.03 |
| Longest Duration NSVT, mean (SD) | 6.6 (4.8)  (n=7)** | 6.8 (4.7)  (n=5)** | 5.2 (3.0) (n=6)** | 0.82 |
| 2^nd^ Degree Heart Block | 0 (0.0) | 0 (0.0) | 0 (0.0) | - |
| 3^rd^ Degree (Complete) Heart Block | 0 (0.0) | 0 (0.0) | 1 (1.7) | 0.73 |
| Pauses | 0 (0.0) | 0 (0.0) | 3 (5.0) | 0.38 |
| Sudden Cardiac Death | 0 (0.0) | 0 (0.0) | 0 (0.0) | - |
| BTKi held/stopped due to arrhythmia | 2 (8.3) | 2 (14.3) | 19 (31.7) | 0.051 |
| Anti-arrhythmic started | 5 (20.8) | 2 (14.3) | 28 (46.7) | 0.016 |
| AF Ablation | 2 (8.3) | 0 (0.0) | 5 (8.3) | 0.53 |
| VT Ablation | 0 (0.0) | 0 (0.0) | 0 (0.0) | - |
| BTKi re-challenged | 1 (4.2) | 0 (0.0) | 1 (1.7) | 0.65 |

Abbreviations: AF, atrial fibrillation; BTKi, Brutons tyrosine kinase inhibitor; PACs, premature atrial contractions; PVCs, premature ventricular contractions; NSVT, non-sustained ventricular tachycardia; SVT, supraventricular tachycardia; VT, ventricular tachycardia. *Except when otherwise specified. **Denominators below listed means denote variables where a different number at risk is considered (eg. denominator reflective of those without prior AF, in incident AF assessments). ***Excludes patients previously on ibrutinib.

**sTable 7C.** Rhythm monitor results for reversible (nemtabrutinib and pirtuobrutinib) next-generation BTKi therapies.

| **Variable, n (%)*** | **Reversible**  **Next-Generation BTKi’s (n=4)**** |
| --- | --- |
| Nemtabrutinib | 3 (75.0) |
| Pirtobrutinib | 1 (25.0) |
| Monitor Duration (days), mean (SD) | 5.8 (5.4) |
| Long-term monitoring (>48 hours), n (%) | 2 (50.0) |
| Any Arrhythmia | 3 (75.0) |
| AF | 0 (0.0) |
| Incident AF | 0 (0.0) |
| AF Burden, mean (SD) | - |
| AF Burden, median (SD) | - |
| High AF Burden (>10%) | 0 (0.0) |
| Moderate AF Burden (5-9.9%) | 0 (0.0) |
| Low AF Burden (<5%) | 4 (100) |
| Ventricular Arrhythmia (excluding PVCs) | 0 (0.0) |
| Ventricular Arrhythmia (including symptomatic or >1% PVCs) | 2 (50.0) |
| PVCs (symptomatic or >1%) | 2 (50.0) |
| PVC Burden (%), mean (SD) | 4.0% (3.0)  (n=2) |
| Non-AF SVTs (excluding PACs) | 1 (25.0) |
| Non-AF SVTs (including symptomatic or >1% PACs) | 2 (50.0) |
| PACs (symptomatic or >1%) | 1 (25.0) |
| Both atrial and ventricular arrhythmias (excluding PAC’s and PVC’s) | 0 (0.0) |
| Longest Duration SVT (beats), mean (SD) | 14.0 (14.0) (n=1) |
| Longest Duration NSVT, mean (SD) | - |
| 2^nd^ Degree Heart Block | 0 (0.0) |
| 3^rd^ Degree (Complete) Heart Block | 0 (0.0) |
| Any Pause | 0 (0.0) |
| Sudden Cardiac Death | 0 (0.0) |
| BTKi held/stopped due to arrhythmia | 0 (0.0) |
| Anti-arrhythmic started | 0 (0.0) |
| AF Ablation | 0 (0.0) |
| VT Ablation | 0 (0.0) |
| BTKi re-challenged | 0 (0.0) |

Abbreviations: AF, atrial fibrillation; BTKi, Brutons tyrosine kinase inhibitor; PACs, premature atrial contractions; PVCs, premature ventricular contractions; NSVT, non-sustained ventricular tachycardia; SVT, supraventricular tachycardia; VT, ventricular tachycardia. *Except when otherwise specified. **Exploratory given only very recent clinical use.

**sTable 8.** Cardiovascular Outcomes following cardiac rhythm monitoring.

| **Variable, n (%)** | **Next-Generation BTKi (n=38)** | **Ibrutinib**  **(n=60)** | ***P*-value** |
| --- | --- | --- | --- |
| Stroke | 2 (5.3) | 4 (6.7) | 0.78 |
| TIA | 1 (2.6) | 5 (8.3) | 0.25 |
| MI | 0 (0.0) | 2 (3.3) | 0.26 |
| New Heart Failure (EF <50%) | 2 (5.3) | 8 (13.3) | 0.20 |
| AF Ablation | 2 (5.3) | 5 (8.3) | 0.57 |
| Hospital Admission for Symptomatic AF | 6 (15.8) | 19 (31.7) | 0.08 |
| Sudden Cardiac Death | 1 (2.6) | 1 (1.7) | 0.74 |
| Cardiovascular Death | 2 (5.3) | 2 (3.3) | 0.64 |
| All-Cause Mortality | 7 (18.4) | 22 (36.7) | 0.054 |

Abbreviations: AF, atrial fibrillation; BTKi, Bruton’s tyrosine kinase inhibitor; EF, ejection fraction; MI, myocardial infarction; TIA, transient ischemic attack.

**sTable 9.** Univariable predictors of major cardiovascular events, by atrial fibrillation burden status, excluding AF events ≤30 days of monitor placement.

| **Variable** | **Hazards Ratio** | **95% Confidence Interval** | ***P*-value** |
| --- | --- | --- | --- |
| High AF Burden (≥10%) | 2.52 | 1.12 – 5.65 | 0.025 |
| Age | 1.04 | 1.00 – 1.08 | 0.045 |
| Male | 0.37 | 0.14 – 0.97 | 0.044 |
| BMI >30 | 1.45 | 0.69 – 3.03 | 0.32 |
| Hypertension | 1.52 | 0.74 – 3.14 | 0.26 |
| Prior AF | 3.06 | 1.31 – 7.11 | 0.010 |
| DM | 0.97 | 0.40 – 2.36 | 0.94 |
| Prior AF Ablation | 0.99 | 0.98 – 1.00 | 0.11 |
| Any Ventricular Arrhythmia | 1.12 | 0.43 – 2.93 | 0.82 |
| PVC/VT >1% Burden | 1.07 | 0.41 – 2.80 | 0.88 |
| PVC/VT >5% Burden | 1.75 | 0.61 – 5.03 | 0.30 |
| PVC/VT >10% Burden | 2.33 | 0.55 – 9.77 | 0.25 |
| PVC/VT >20% Burden | 3.23 | 0.43 – 24.1 | 0.25 |
| Total duration of (any) BTKi Therapy at time of rhythm monitor, months | 1.01 | 0.996 – 1.02 | 0.15 |
| Total duration of ibrutinib at time of rhythm monitor, months | 0.99 | 0.98 – 1.01 | 0.62 |
| Total duration of next-generation BTKi at time of rhythm monitor | 0.99 | 0.98 – 1.02 | 0.996 |
| Primary Malignancy of CLL | 0.70 | 0.26 – 1.92 | 0.49 |
| Smoker (current or former) | 2.59 | 1.26 – 5.30 | 0.009 |
| Prior Chemotherapy | 1.14 | 0.54 – 2.37 | 0.73 |
| CHF | 5.96 | 1.80 – 19.7 | 0.003 |
| Prior Immunomodulatory Therapy | 1.62 | 0.70 – 3.76 | 0.26 |
| LVEF | 0.97 | 0.89 – 1.07 | 0.59 |
| LVEF Low (≤ 50%) | 2.52 | 0.60 – 10.6 | 0.21 |
| Total Duration of (any) BTKi Therapy less than 1 year | 0.27 | 0.08 – 0.89 | 0.031 |
| Next Generation BTKi* | 1.01 | 0.46 – 2.23 | 0.97 |
| Beta-blocker | 1.36 | 0.58 – 3.17 | 0.48 |
| Calcium channel blocker | 1.25 | 0.48 – 3.25 | 0.65 |
| Cardio-Oncologist | 0.64 | 0.22 – 1.83 | 0.41 |
| Electrophysiologist | 3.23 | 1.59 – 6.55 | 0.001 |
| Any cardiologist | 3.61 | 0.49 – 26.5 | 0.21 |

Abbreviations: AF, atrial fibrillation; BMI, body mass index; BTKi, Bruton's Tyrosine Kinase inhibitor; CHF, congestive heart failure; CLL, chronic lymphocytic leukemia; DM, diabetes mellitus; LVEF, left ventricular ejection fraction; PVC, premature ventricular contractions; VT, ventricular tachycardia. *Next-generation BTKi includes acalabrutinib and non-covalent drugs (here pirtobrutinib, nemtabrutinib).

**sTable 10.** Univariable predictors of all-cause mortality, by atrial fibrillation burden status.

| **Variable** | **Hazards Ratio** | **95% Confidence Interval** | ***P*-value** |
| --- | --- | --- | --- |
| High AF Burden (≥10%) | 3.18 | 1.47 – 6.88 | 0.003 |
| Age | 1.07 | 1.02 – 1.12 | 0.004 |
| Male | 1.39 | 0.64 – 3.00 | 0.41 |
| BMI >30 | 1.52 | 0.71 – 3.28 | 0.29 |
| Hypertension | 1.00 | 0.48 – 2.08 | >0.99 |
| Prior AF | 1.70 | 0.65 – 4.47 | 0.28 |
| DM | 0.90 | 0.34 – 2.36 | 0.83 |
| Prior AF Ablation | 1.89 | 0.44 – 7.99 | 0.39 |
| PVC/VT >1% Burden | 1.60 | 0.65 – 3.94 | 0.31 |
| PVC/VT >5% Burden | 2.12 | 0.73 – 6.11 | 0.17 |
| PVC/VT >10% Burden | 0.98 | 0.13 – 7.27 | 0.99 |
| PVC/VT >20% Burden | 4.24 | 0.56 – 32.1 | 0.16 |
| PVC/VT >1% Burden | 1.60 | 0.65 – 3.94 | 0.31 |
| Total Duration of (any) BTKi Therapy at time of rhythm monitor, months | 1.01 | 1.00 - 1.03 | 0.15 |
| Total duration of ibrutinib therapy at time of rhythm monitor | 1.01 | 0.99-1.02 | 0.37 |
| Total duration of next-generation BTKi at time of rhythm monitor | 1.01 | 0.99-1.02 | 0.48 |
| Primary Malignancy of CLL | 0.57 | 0.23 - 1.41 | 0.22 |
| Smoker (current or former) | 1.90 | 0.91 – 3.95 | 0.09 |
| Prior Immunomodulatory therapy | 0.70 | 0.27 – 2.21 | 0.62 |
| CHF | 1.76 | 0.41 – 7.49 | 0.45 |
| Prior Chemotherapy | 1.12 | 0.50 – 2.51 | 0.77 |
| LVEF | 1.004 | 0.93 – 1.09 | 0.92 |
| Total Duration of (any) BTKi Therapy less than 1 year | 1.35 | 0.31 – 5.84 | 0.69 |
| Next Generation BTKi* | 1.30 | 0.52 – 3.22 | 0.58 |
| Beta-blocker | 1.56 | 0.66 – 3.69 | 0.31 |
| Calcium channel blocker | 1.45 | 0.50 – 4.18 | 0.49 |
| Cardio-Oncologist | 0.71 | 0.25 – 2.04 | 0.52 |
| Electrophysiologist | 1.38 | 0.65 – 2.94 | 0.41 |
| Any cardiologist | 2.88 | 0.39 – 21.2 | 0.30 |

Abbreviations: AF, atrial fibrillation; BMI, body mass index; BTKi, Bruton's Tyrosine Kinase inhibitor; CHF, congestive heart failure; CLL, chronic lymphocytic leukemia; DM, diabetes mellitus; LVEF, left ventricular ejection fraction; PVC, premature ventricular contractions; VT, ventricular tachycardia. *Next-generation BTKi includes acalabrutinib and non-covalent drugs (here pirtobrutinib, nemtabrutinib)

**sTable 11A.** Multivariable predictors of major cardiovascular events, by atrial fibrillation burden status, excluding events within ≤ 30 days of monitor placement.

| **Variable** | **Hazards Ratio** | **95% Confidence Interval** | ***P*-value** |
| --- | --- | --- | --- |
| High AF Burden (≥10%) | 2.95 | 1.23 – 7.04 | 0.015 |
| Prior AF | 3.25 | 1.33 – 7.90 | 0.009 |
| CLL | 0.70 | 0.26 – 1.92 | 0.49 |
| Total Duration of (any) BTKi Therapy*, months | 1.02 | 1.00 – 1.03 | 0.015 |

Abbreviations: AF, atrial fibrillation; BTKi, Bruton’s Tyrosine Kinase inhibitor; CLL, chronic lymphocytic leukemia. *At time of rhythm monitoring.

**sTable 11B.** Additional multivariable analyses for predictors of major cardiovascular events, by atrial fibrillation burden status.

| **Variable** | **Hazards Ratio** | **95% Confidence Interval** | ***P*-value** |
| --- | --- | --- | --- |
| High AF Burden (≥10%) | 2.57 | 1.06 – 6.25 | 0.037 |
| Prior AF | 2.11 | 0.83 – 5.37 | 0.12 |
| DM | 1.08 | 0.43 – 2.75 | 0.87 |
| Prior Ablation | 3.86 | 1.04 – 14.3 | 0.043 |
| Total Duration of (any) BTKi Therapy*, months | 1.01 | 0.998 – 1.02 | 0.11 |

Abbreviations: AF, atrial fibrillation; BTKi, Bruton's Tyrosine Kinase inhibitor; DM, diabetes mellitus. *At time of rhythm monitoring.

**sTable 12.** Additional multivariable analyses for predictors of all-cause mortality, by atrial fibrillation burden status.

| **Variable** | **Hazards Ratio** | **95% Confidence Interval** | ***P*-value** |
| --- | --- | --- | --- |
| High AF Burden (≥10%) | 3.26 | 1.39 – 7.62 | 0.006 |
| Prior AF | 1.45 | 0.51 – 4.12 | 0.49 |
| DM | 1.29 | 0.46 – 3.61 | 0.63 |
| Total Duration of (any) BTKi Therapy*, months | 1.01 | 1.00 – 1.03 | 0.10 |

Abbreviations: AF, atrial fibrillation; BTKi, Bruton’s Tyrosine Kinase inhibitor; DM, diabetes mellitus. *At time of rhythm monitoring.

**sFigure 1:** Study Cohort Diagram. From a registry of all patients with hematologic malignancies treated with BTKi’s (including ibrutinib, acalabrutinib, pirtubrutinib, or any other early phase BTKi agent), over an 11-year period, those with available ambulatory cardiac rhythm monitors (or mobile telemetry) were included. AF, atrial fibrillation; BTKi, Brutons tyrosine kinase inhibitor; PVC, premature ventricular contraction; SVT, supraventricular tachycardia; VA, ventricular arrhythmia. *AF burden, % of beats in AF over a 24-hour period.


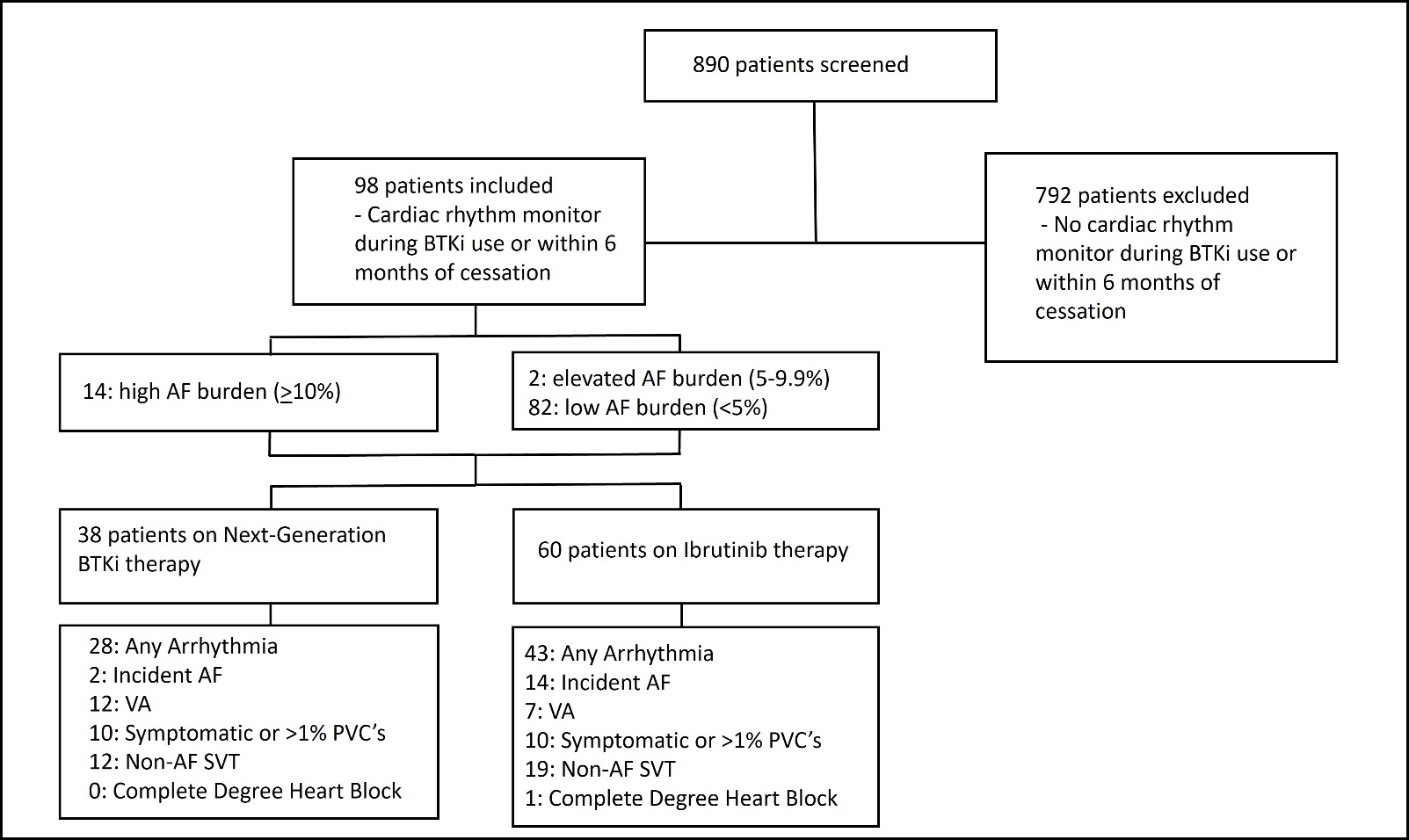


**sFigure 2A. Cumulative Incidence of MACE by degree of AF burden following BTKi treatment initiation.** High AF burden defined as ≥ 10% of all beats over 24 hours being AF. This analysis excluded AF events that occurred within 30 days of ambulatory rhythm monitoring from MACE definition. AF, atrial fibrillation; BTKi, Bruton’s tyrosine kinase inhibitor; MACE, major cardiovascular events.

**
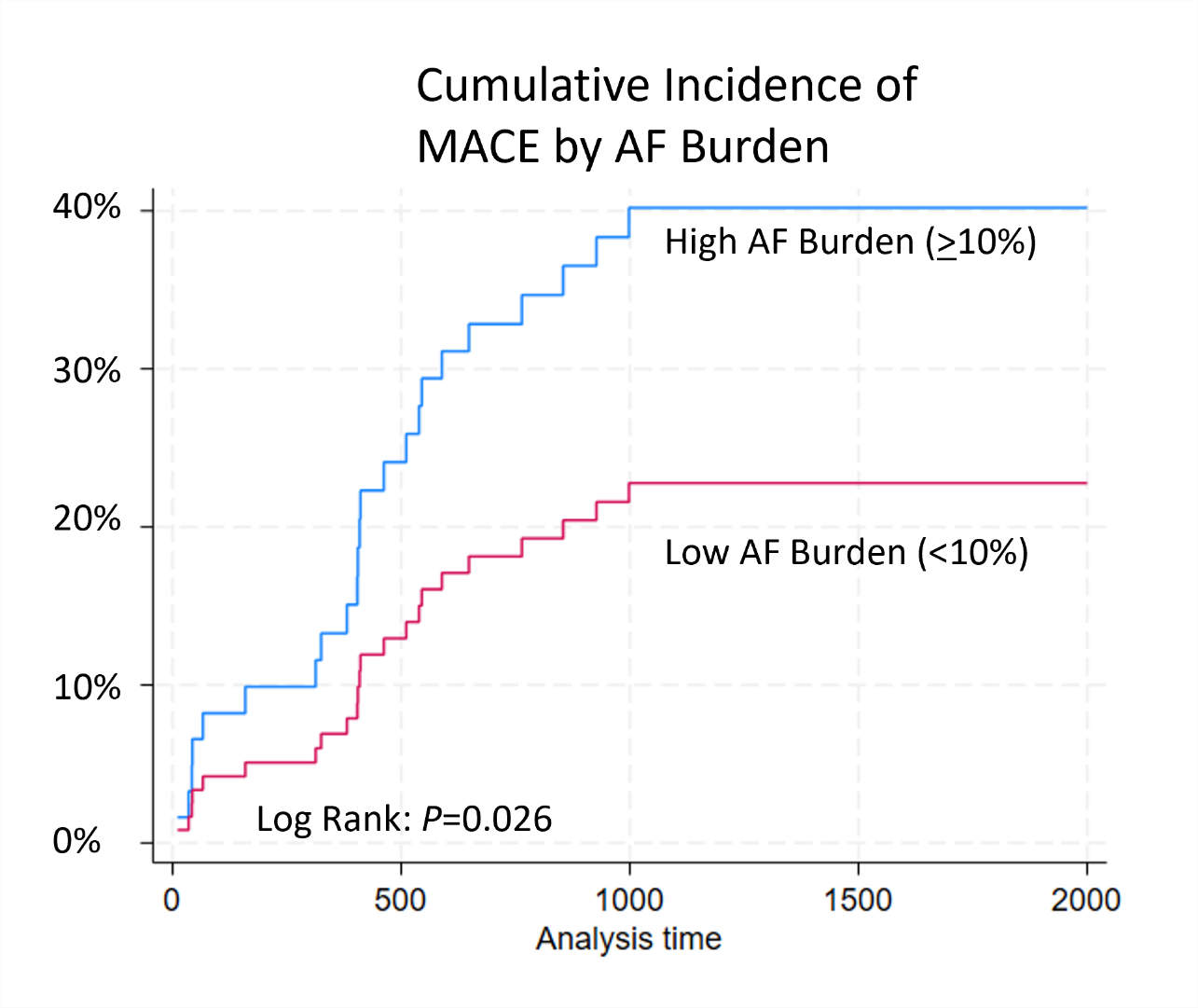
**

**sFigure 2B. MACE-free survival by degree of AF burden following BTKi treatment initiation.** High AF burden defined as ≥ 10% of all beats over 24 hours being AF, associates with worse MACE-free survival. This analysis excluded AF events that occurred within 30 days of ambulatory rhythm monitoring from MACE definition. AF, atrial fibrillation; BTKi, Bruton’s tyrosine kinase inhibitor; MACE, major cardiovascular events.

**
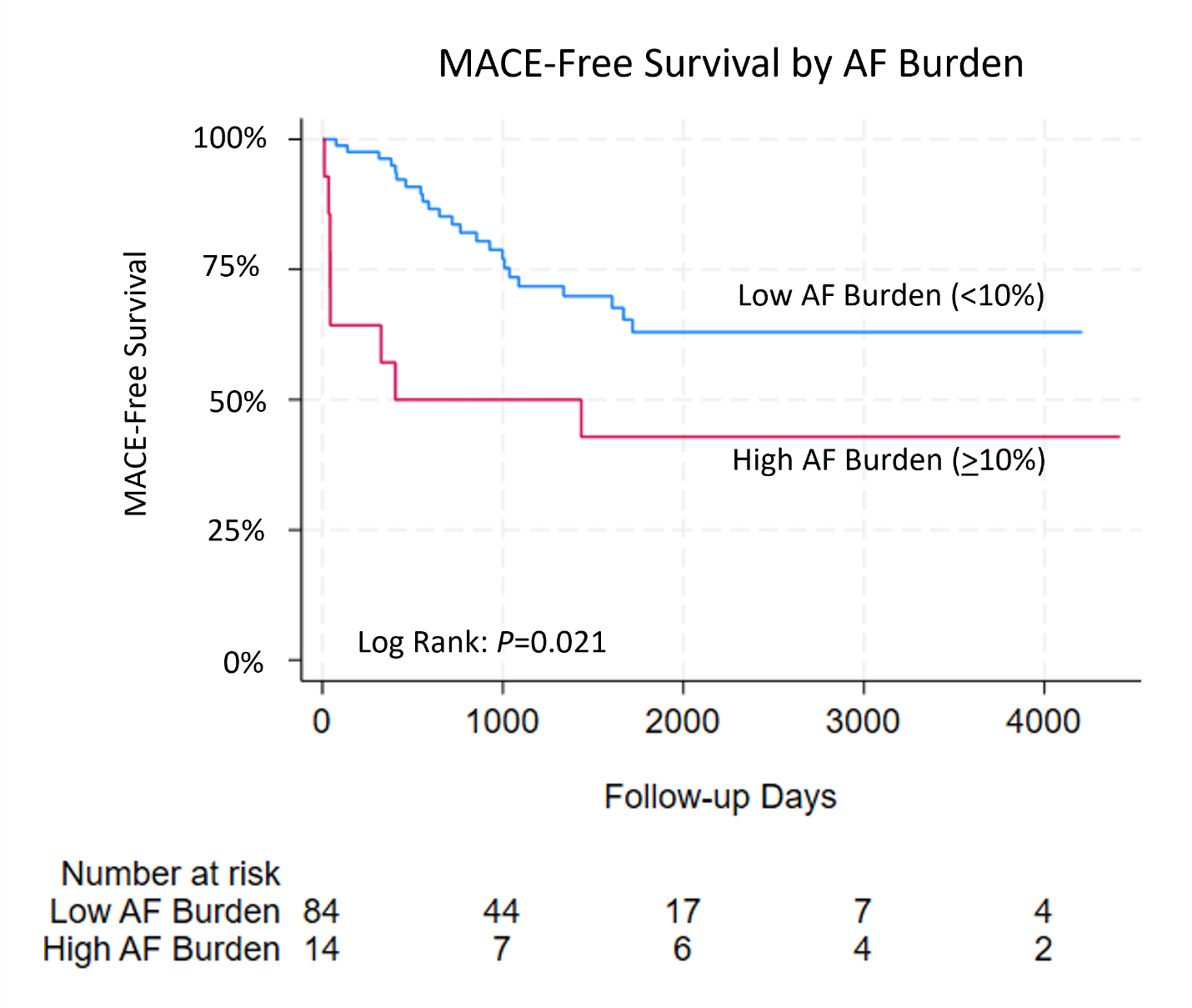
**

**Log-rank *P*-value (Competing Risks Regression) = 0.0208**

**sFigure 3.** Receiver operator curve (ROC) analysis for the relation between atrial fibrillation burden and subsequent major cardiovascular events (MACE) following BTKi therapy initiation.

**
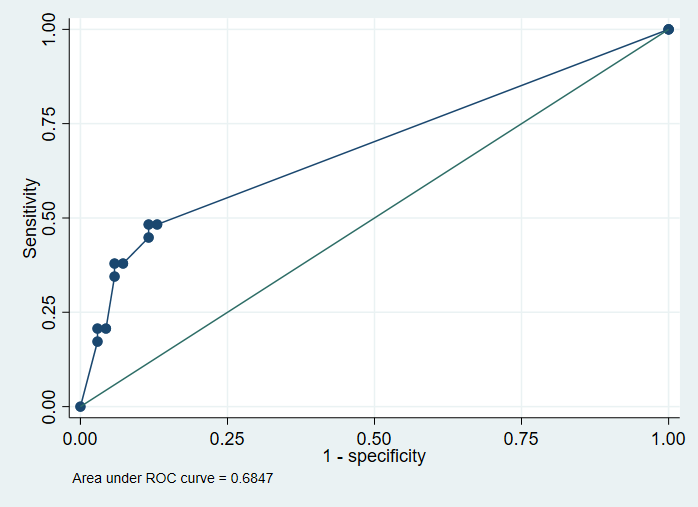
**
